# Non-allergic rhinitis: a case report and review

**DOI:** 10.1186/1476-7961-8-1

**Published:** 2010-02-03

**Authors:** Cyrus H Nozad, L Madison Michael, D Betty Lew, Christie F Michael

**Affiliations:** 1 Division of Clinical Immunology, University of Tennessee Health Science Center, 50 North Dunlap St, RM 401 WPT, Memphis, TN, USA; 2 Department of Neurosurgery, University of Tennessee Health Science Center, Memphis, TN, USA; 3 Semmes-Murphey Neurologic and Spine Center, 6325 Humphreys Blvd, Memphis, TN, USA

## Abstract

Rhinitis is characterized by rhinorrhea, sneezing, nasal congestion, nasal itch and/or postnasal drip. Often the first step in arriving at a diagnosis is to exclude or diagnose sensitivity to inhalant allergens. Non-allergic rhinitis (NAR) comprises multiple distinct conditions that may even co-exist with allergic rhinitis (AR). They may differ in their presentation and treatment. As well, the pathogenesis of NAR is not clearly elucidated and likely varied. There are many conditions that can have similar presentations to NAR or AR, including nasal polyps, anatomical/mechanical factors, autoimmune diseases, metabolic conditions, genetic conditions and immunodeficiency. Here we present a case of a rare condition initially diagnosed and treated as typical allergic rhinitis vs. vasomotor rhinitis, but found to be something much more serious. This case illustrates the importance of maintaining an appropriate differential diagnosis for a complaint routinely seen as mundane. The case presentation is followed by a review of the potential causes and pathogenesis of NAR.

## Introduction

The term rhinitis can be used to describe many distinct entities with varying pathogeneses, despite similar presentations. Generally, rhinitis is considered allergic if significant inhalant allergy is diagnosed and is considered non-allergic when symptomatology is perennial or periodic and not IgE mediated. Thus non-allergic rhinitis (NAR) comprises a mixed bag of conditions ranging from vasomotor rhinitis (VMR) to hormonally induced rhinitis.

Overall, rhinitis results in significant cost to the world population. In 2002, the direct and indirect costs for allergic rhinitis (AR) were estimated to be $7.3 billion and $4.28 billion, respectively [1]. Given that an estimated 1 in 3 patients with rhinitis are diagnosed with NAR, with 19 million people in the United States alone, it is reasonable to conclude that NAR also results in a significant economic burden [2-4].

NAR is a condition primarily seen in adulthood with 70% of cases developing after the age of 20. There is a greater prevalence among females compared to males [5,6], and the overall prevalence of NAR in industrialized countries has ranged from 20-40% [7]. The following case presentation is an example of a patient with typical NAR symptoms who fits the epidemiological profile, but who presented atypically, failed to respond to standard therapy and was subsequently found to have a much more serious underlying condition.

## Case Report

A 52 year old African American female presented to our outpatient allergy clinic with a chief complaint of profuse "runny nose" for 1 week. She initially attributed the rhinorrhea to prolonged moth ball exposure in her small office space. Prior to her visit she had been seen by her primary care provider and in the emergency department. Both treated her for allergic rhinitis vs. vasomotor rhinitis with intranasal corticosteroids and oral antihistamines. She also complained of a diffuse headache, imbalance, cough and right ear fullness. She denied any history of previous rhinitis symptoms, eye symptoms or sneezing. Her past medical history was significant for hypertension, hypercholesterolemia, diabetes mellitus (poorly controlled) and excision of a benign scalp lesion at age 18. Family history was significant for a father and eight siblings with allergic rhinitis and one brother with asthma. Her social history was significant for pet and tobacco smoke exposure. She denied any illicit drug use. Review of systems was negative for trauma, fevers, chills, weight loss, night sweats, shortness of breath, chest pain, nausea, vomiting or diarrhea; and positive for, diffuse headaches, unsteadiness and a cough productive of clear sputum that was worse when supine.

On physical exam her vital signs were: BP 172/86, P 89, R 18, T 36.2 C, W 95 kg. Generally, she presented to us holding a rag over her nose, coughing, but not in distress. She had a normocephalic/atraumatic head, tympanic membranes were clear, and conjunctivae were clear. She had copious clear nasal discharge from the right naris with a continual drip. The nasal mucosa was otherwise moist and mildly erythematous; with slightly atrophic turbinates. There was no chest deformity. The lungs were clear to auscultation without wheezes, rubs or rhonchi. Cardiovascularly, she had a regular rate and rhythm without murmurs, rubs or gallops. The abdomen was soft, non-tender/non-distended and no masses were palpated. Skin exam revealed no lesions. Extremity examination did not reveal any cyanosis, clubbing or edema. Lastly she had no enlarged cervical lymph nodes.

**Figure 1:**
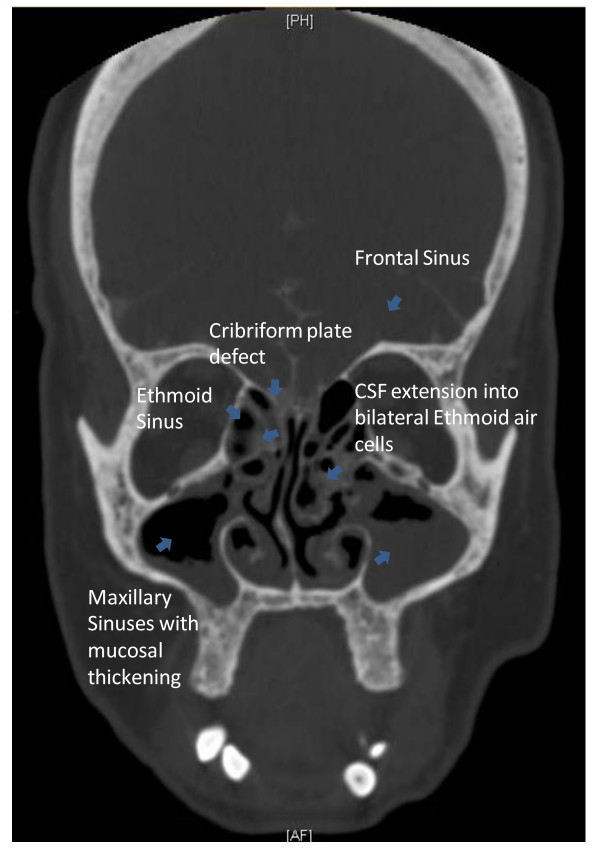
**Preoperative CT scan of the head**. A preoperative CT scan of the head with intrathecal contrast was done to localize the CSF leak. The frontal view is shown here. The scan shows a defect in the cribriform plate with confluent CSF extension into several bilateral ethmoid air cells, evidenced by the higher intensity signal of the contrast. Secondarily, there is mucosal thickening in the ethmoid and maxillary sinuses.

A sample of her nasal rhinorrhea was sent to the lab to analyze for beta-2 transferrin. She was then sent for a stat CT scan of her head. The patient's rhinorrhea sample was positive for beta-2 transferrin, consistent with a cerebrospinal fluid leak (CSF). The CT scan showed mucosal thickening of the ethmoid sinuses and nasal septal deviation, but no intracranial findings to explain CSF leak. Consultation with Neurosurgery and ENT were made. When the leak failed to resolve spontaneously, she underwent a CT cisternogram to localize the leak. A defect was identified in the cribriform plate bilaterally with CSF extension into several bilateral ethmoid air cells, Figure 1. She underwent an endoscopic ethmoidotomy and repair of her cerebrospinal fluid leak by ENT. After surgery, neurosurgery placed a lumbar drain for 72 hours to divert the CSF in order for the repair to properly seal. Repeat CT cisternogram 1 month later no longer showed evidence of CSF leak.

## Cerebrospinal Rhinorrhea Etiology, Diagnosis and Treatment

**Figure 2:**
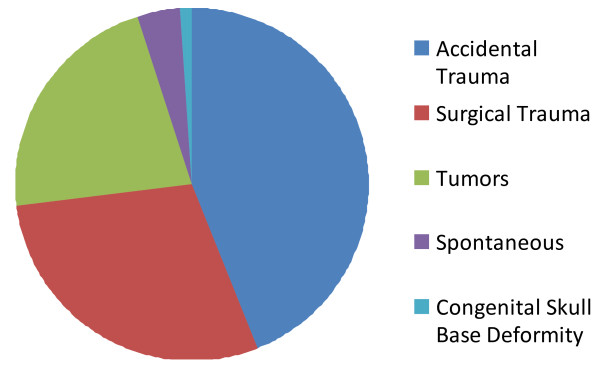
**Etiology of CSF Rhinorrhea**. CSF leak can be attributed to the following causes: Accidental trauma from a closed head injury (44%), surgical trauma (29%), tumors (22%), spontaneously due to increased intracranial pressure (ICP) (3-4%, but in some series are as high as 15-20%) and congenital skull base deformities (rare)[8-10].

CSF is produced in the choroid plexus at a rate of 350-500 ml per day. The total adult volume is 90-150 ml. This volume is turned over 3-5 times per day via continual absorption by the arachnoid villa. The etiologies of CSF leak include accidental trauma from a closed head injury (44%), surgical trauma (29%), tumors (22%), congenital skull base deformities (rare), and spontaneous leak (3-4%, but in some series are as high as 15-20%) due to increased intracranial pressure (ICP) [8-10], Figure 2.

As in our case, the diagnosis starts with identification of the rhinorrhea as CSF. The fluid is analyzed for beta-2 transferrin, which is formed by the conversion of beta-1 transferrin into beta-2 transferrin by cerebral neuraminidase [11]. Beta-2 transferrin is only found in the CSF, vitreous humor of the eye and perilymph. Next, the site of the leak must be identified. A high resolution CT scan of the head can evaluate the paranasal sinuses as well as the skull base. Adding intrathecal contrast can allow for better localization of a leak. If a patient has an inactive or intermittent leak, they may have a negative CT scan. CT scan has a sensitivity ranging from 48 to 96% [11,12]. Magnetic resonance cisternography is another method of localization of CSF leaks and has a sensitivity of 92-100%. MRI is also used as an adjunct test for further characterization of meningoencephalocele, if present [13].

Patients with a spontaneous CSF leak, due to an associated increase in intracranial pressure can have characteristic findings. These findings are, a broadly thinned and attenuated skull base, dehiscence of the ethmoid roof, arachnoid pits (from bony impressions from arachnoid villa), pneumatization of the lateral sphenoid sinus recess and meningoencephalocele formation [14-16]. The most common sites for a leak in these patients include, the lateral recess of the sphenoid sinus and the ethmoid roof or cribriform [11].

Treatment can consist of a traditional open craniotomy with repair of the defect. This approach has a success rate of 70-80% [17,18]. Newer endoscopic repairs have shown greater success, with rates >90% and avoid the morbidity associated with craniotomy [19]. For patients with elevated intracranial pressure, cerebrospinal fluid diversion procedures (i.e. ventriculoperitoneal shunt or lumboperitoneal shunt) may be needed to successfully stop the egress of fluid. Lastly, some patients will have spontaneous closure of their leak.

## Non-Allergic Rhinitis Classification

NAR consists of multiple entities, some of which share components of both AR and NAR. One way of further classifying these conditions distinguishes pure non-allergic rhinitis (without any component of AR), occupational rhinitis and other rhinitis syndromes. The pathogeneses of these different conditions is not clearly defined and there are many proposed mechanisms.

## Non-Allergic Rhinitis

Vasomotor or idiopathic rhinitis (VMR) is the most commonly diagnosed form of NAR, accounting for ~60% cases in one series [5]. VMR is characterized by sporadic or persistent nasal symptoms that are trigged by: strong smells, cold air, changes in temperature, humidity, barometric pressure, strong emotions, alcohol and changes in hormone levels. Diagnosis is made clinically and onset is typically in adulthood. Intranasal corticosteroids and intranasal antihistamines are the mainstay of therapy.

Gustatory rhinitis typically occurs after ingestion of heated foods, spicy foods or alcohol. Profuse rhinorrhea may be vagally mediated, due to a food allergy and/or other undefined mechanisms. Treatment consists of intranasal ipratropium bromide as needed.

Infectious rhinitis may be acute or chronic in nature. Symptoms include nasal congestion, mucopurulent nasal discharge, frontal headache, olfactory disturbances, postnasal drainage and cough. Viral infections account for as many as 98% of acute infectious rhinitis in young children [20]. Treatment is symptomatic, unless a bacterial cause is suspected and topical antibacterial agents can be used in some cases.

Non-allergic rhinitis with eosinophilia syndrome (NARES) usually develops in adulthood and is characterized by year round nasal symptoms such as, profuse rhinorrhea and nasal congestion. These patients have negative allergy skin testing and normal serum IgE levels. Classically, nasal smears have greater than 5% to greater than 20% eosinophils [21]. Many of these patients may develop aspirin (ASA) sensitivity, sinusitis, nasal polyps, and asthma. These patients are also at increased risk of developing obstructive sleep apnea [22].

There is also a variant of NARES called blood eosinophilia-nonallergic rhinitis syndrome (BENARES). These patients share the same characteristics as patients with NARES except they lack nasal eosinophilia and instead have elevated serum eosinophil levels. Intranasal corticosteroids are the treatment of choice for both NARES and BENARES [5,23].

## Occupational Rhinitis

Occupational rhinitis can develop after exposures, typically in a work environment, and may be allergic or non allergic in nature. There are four types of occupational rhinitis. The first type is annoyance rhinitis, which is purely subjective, typically fragrance-induced, and occurs without evidence of nasal inflammation. The second type is irritant-induced rhinitis, and manifests as inflammation of the nasal mucosa without apparent immunologic or allergic basis [24].

The third type is corrosive rhinitis, following exposure to high concentrations of irritating and soluble chemical gases. Examples include ammonia and pesticides. The fourth and final type is allergic rhinitis, with evidence of an IgE mediated reaction to an occupational exposure. An example would be latex allergy in a healthcare worker. Treatment in all of these different rhinitis types consists of nasal saline to remove particulate matter, nasal corticosteroids, nasal antihistamines and avoidance. The exception is corrosive rhinitis, in which case avoidance is the only option.

## Other Rhinitis Syndromes

Hormonally induced rhinitis, includes menstrual cycle related rhinitis and rhinitis of pregnancy. Rhinitis of pregnancy typically begins in the 2^nd ^trimester with severe congestion and resolves about 2 weeks postpartum [25]. Treatment consists of saline nose spray or nasal lavage. Exercise may lead to physiologic nasal vasoconstriction. External nasal dilators (e.g. strips applied to the bridge of the nose to buttress open the nares) may be effective for patients with pregnancy-related nocturnal nasal congestion. Typically, rhinitis of pregnancy requires no specific pharmacologic intervention and intranasal glucocorticoids have not been shown to be effective. There is anecdotal evidence to support the use of nasal ipratropium and pseudoephedrine; therefore, these agents may be useful in patients who are particularly symptomatic. However, pseudoephedrine should be avoided in the first trimester of pregnancy and in women with hypertension.

Rhinitis medicamentosa is characterized by severe nasal congestion, due to a rebound effect from overuse of topical decongestants, such as oxymetazoline, an imidazole, and phenylephrine, a sympathomimetic amine. Treatment consists of topical nasal corticosteroids and/or oral corticosteroids, with progressive withdrawal of the topical decongestant over 3-7 days. Other medications can lead to nasal symptoms as a side effect, usually congestion. Examples include ACE inhibitors, β-blockers, aspirin and NSAIDs.

Atrophic rhinitis is characterized by progressive nasal atrophy, mucosal colonization with Klebsiella ozaenae or other organisms, and a foul smelling nasal discharge. It can be seen as a post surgical complication, i.e. status post turbinectomy. Treatment consists of nasal lavage, lubrication and topical antibiotics are used for mucopurulent secretions lasting beyond 2 days. Oral antibiotics can also be used for acute infections. Surveillance rhinoscopy should be performed at least twice a year if the patient remains symptomatic [26]. The clinical presentations and treatments for non-allergic rhinitis are summarized in Table 1.

**Table 1:** Types of non-allergic rhinitis, clinical presentation and treatments

RHINITIS	CLINICAL PRESENTATION	TREATMENT
**Non-Allergic Rhinitis:**		
Vasomotor Rhinitis	Typically adult onset, sporadic or persistent nasal symptoms trigged by strong smells, cold air, changes in temperature, humidity, barometric pressure, strong emotions, alcohol and changes in hormone levels.	Intranasal corticosteroids and/or intranasal antihistamines are the mainstay of therapy

Gustatory Rhinitis	Profuse rhinorrhea after ingestion of heated foods, spicy foods or alcohol.	Intranasal ipratropium bromide as needed.

Infectious Rhinitis	Nasal congestion, mucopurulent nasal discharge, frontal headache, olfactory disturbances, postnasal drainage and cough.	Symptomatic treatment for viral infections. Topical antibacterial agents, i.e. mupirocin, for suspected bacterial infections.

Non-allergic rhinitis with eosinophilia syndrome (NARES)	Typically adult onset. Individuals experience year round profuse rhinorrhea and nasal congestion. These patients have negative allergy skin testing and normal serum IgE levels.	Intranasal corticosteroids.

**Occupational Rhinitis: **[54-58]		
Annoyance	Patients report rhinitis symptoms that are purely subjective after occupational exposures. Symptoms are typically fragrance-induced, and occur without evidence of nasal inflammation.	Avoidance of triggers, nasal saline, nasal corticosteroids and nasal antihistamines.

Irritant	Rhinitis symptoms after occupational exposure to irritants (e.g. cigarette smoke), and these patients have objective findings such as inflammation of the nasal mucosa without apparent immunologic or allergic basis.	Avoidance of triggers, nasal saline, nasal corticosteroids and nasal antihistamines.

Corrosive	Rhinitis symptoms that occur after occupational exposure, to high concentrations of irritating and soluble chemical gases that in turn cause nasal inflammation which can break down and ulcerate the nasal mucosa.	Avoidance of the inciting agent.

Allergic	Rhinitis symptoms due to an IgE mediated reaction to an occupational exposure.	Avoidance of triggers, nasal saline, nasal corticosteroids and nasal antihistamines.

**Other Rhinitis Syndromes:**[60-63]		
Hormonally induced Rhinitis	Includes menstrual cycle related rhinitis and rhinitis of pregnancy. Rhinitis of pregnancy typically begins in the 2nd trimester with severe congestion and resolves about 2 weeks postpartum[25].	Usually requires no specific pharmacologic intervention and treatment consists of saline nose spray or nasal lavage. External nasal dilator may be effective for patients with pregnancy-related nocturnal nasal congestion. Intranasal glucocorticoids have not been shown to be effective.

Rhinitis Medicamentosa	Severe nasal congestion, due to a rebound effect from overuse of topical decongestants, such as oxymetazoline and phenylephrine.	Topical nasal corticosteroids and/or oral corticosteroids, with progressive withdrawal of the topical decongestant over 3-7 days.

**Atrophic Rhinitis:**[66-68]		
Primary Atrophic Rhinitis	Progressive nasal atrophy, mucosal colonization with Klebsiella ozaenae or other organisms, and a foul smelling nasal discharge. It can be seen as a post surgical complication, i.e. status post turbinectomy.	Nasal lavage, lubrication and topical antibiotics are used for mucopurulent secretions lasting beyond 2 days. Oral antibiotics can also be used for acute infections. Surveillance rhinoscopy should be performed at least twice a year if the patient remains symptomatic[26].
Secondary Atrophic Rhinitis		

Additional treatments for the various forms of rhinitis do exist. The most common or first line therapies are listed here.

## Pathogenesis

**Figure 3:**
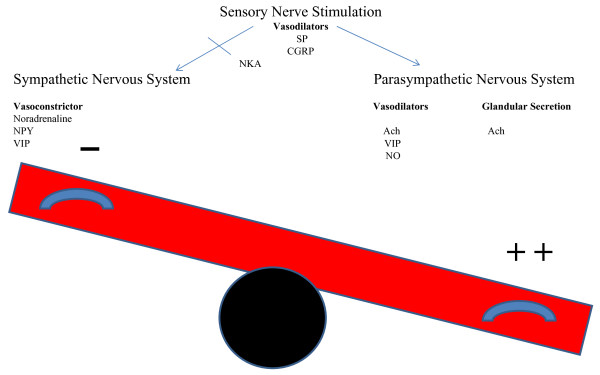
**Balance of autonomic inputs into nasal mucosa of patients with vasomotor rhinitis (VMR)**. Central nervous system stimulation causes release of tachykinins, substance P (SP), calcitonin gene-related peptide (CGRP) and neurokinin A (NKA) that result in inhibition of the sympathetic nervous system and shift the balance towards the parasympathetic response. Noradrenaline and neuropeptide tyrosine (NPY) cause vasoconstriction and relief of nasal congestion. Vasodilators acetylcholine (Ach), vasoactive intestinal peptide (VIP) and nitric oxide (NO) cause nasal congestion and glandular secretion [33].

The mechanisms underlying VMR are poorly understood. Generally it is thought to arise from an imbalance of autonomic input into the nasal mucosa. An initial stimulus results in nasal congestion and/or rhinorrhea induced by tachykinins from the central nervous system, which also inhibit sympathetic mediators and thus further enhance the parasympathetic response [27,28], see Figure 3.

Tai and Baraniuk suggested that sensory C-fiber stimulation leads to release of substance P (SP) and calcitonin gene-related peptides (CGRP). This in turn leads to increased plasma excretion and glandular secretion (via acetylcholine and muscarinic receptors) manifesting as pain and stuffiness [29]. Schierhorn et al. have shown that ozone increases nasal mucosal levels of SP and neurokinin A (NKA) [30]. NKA is also known to have a similar structure and function to SP, resulting in vascular smooth muscle contraction. Therefore, sensory stimulation causes release of SP, CGRP, NKA with subsequent nasal congestion and pain.

Groneberg et al. found elevated levels of neuropeptide tyrosine (NPY), vasoactive intestinal peptide (VIP), and SP in patients with irritant rhinitis caused by cigarette smoke exposure, as compared to controls [31]. NPY is primarily distributed around blood vessels and intranasal administration of NPY has been shown to cause nasal vasoconstriction and a decrease in nitric oxide levels [32]. Noradrenaline also acts to vasoconstrict nasal mucosal blood vessels [33]. VIP is an inhibitory neurotransmitter that can cause vasodilatation and hypersecretion and thus congestion and rhinorrhea. Furthermore, Acetylcholine (Ach) release leads to glandular secretion and vasodilation [33].

The significance of the free radical, nitric oxide (NO), in the context of rhinitis has been examined. NO is generated by nitric oxide synthase (NOS), which requires nicotinamide adenine dinucleotide phosphate (NADPH) as a cofactor. NO is present only transiently, therefore its presence and activity are inferred by the measure of NADPH diaphorase levels in tissue, which correlate with NOS activity. In patients with VMR, epithelial damage correlates with increased NADPH diaphorase activity and thus increased NO activity. Collectively, these data suggest that NO, which has known cytotoxic effects, can cause epithelium damage. This damage could result in impaired mucociliary clearance, loss of tight junctions and basement membrane damage. This would in turn allow for increased reactivity of afferent trigeminal fibers, secretory and vascular reflexes resulting in the constellation of symptoms seen in VMR [34,35].

Analysis of nasal wash protein samples have shown that the total protein and albumin concentration is higher in patients with AR as opposed to NAR and controls, who had the lowest concentrations. A 26 kDa protein, isolated from nasal lavage, was found to be significantly elevated in patients with AR as compared to patients with NAR and controls. Although this protein is not well defined, it is believed to originate from the nasal glands because it is produced in normal subjects upon nasal provocation with pilocarpine. It is thought that this may be used to differentiate AR from NAR [36,37].

Trauma has also been implicated as a cause of VMR [38]. Studies by Giannessi et al. have shown that in patients with VMR, there was ciliary loss, absence of tight junctions, marked distension of the intercellular space, and loss of goblet cells [34].

IgE may also play a role in non-allergic rhinitis. Patients with house dust mite sensitivity by history, negative inhalant allergy skin testing and RAST (radioallergosorbent test), have been found to produce specific IgE antibodies in nasal secretions, but not in serum, upon nasal provocation with house dust mite antigen [39].

A percentage of NAR patients have been found to have increased numbers of nasal mucosal mast cells and eosinophils. These IgE expressing cells further support the role of a local allergic disease process in NAR [40,41]. Furthermore, labeled grass pollen has been shown to be bound by mast cells in patients with NAR and negative allergy inhalant testing [42]. Lastly, first line treatment is usually topical corticosteroids lending further credence to an underlying inflammatory immune mechanism.

Thromboxane A2 (TXA2) is a potent inflammatory mediator. It binds via the TXA2 receptor which is a G-protein coupled receptor. TXA2 receptor agonists have been shown to increase nasal airway resistance and nasal vascular permeability in guinea pigs [43]. Shirasaki et al. suggested that one of the isoforms of the TXA2 receptor, TP alpha may play a role in allergic and non-allergic rhinitis. TP alpha expression has been found in human inferior turbinate tissue, specifically on the smooth muscle layers of the venous sinusoids, arterioles, epithelial cells and submucosal glands in human nasal mucosa from patients with AR and NAR. TXA 2 receptor antagonists have been shown to reduce allergen induced nasal mucosal swelling in patients with AR. Lastly, TXA2 receptor agonists have been shown to induce leukocyte adhesion to vascular walls, suggesting a role of TXA2 in the inflammatory cascade [44].

In patients with gustatory rhinitis, Raphael et al. have shown that increased levels of albumin and total protein (without altering the ratio of albumin to total protein), are identified in the nasal secretions of patients undergoing a food challenge. Furthermore, this effect can be blunted by intranasal atropine, suggesting that atropine-inhibitable muscarinic receptors play a large role in the pathogenesis [45].

The pathogenesis of NARES is controversial and poorly understood. One theory suggests that irritants such as passive smoke exposure, induces a localized allergic inflammatory response [46]. NARES may also represent an early stage of aspirin sensitivity [47]. Lastly, there is a proposed mechanism, that eosinophils may directly damage nasal epithelial cells and lead to protracted mucociliary clearance [48-53].

The pathogenesis of occupational rhinitis is based on the etiology of the rhinitis. Annoyance rhinitis does not show any evidence of nasal mucosal inflammation. Irritant-induced rhinitis triggers include tobacco smoke exposure and symptoms are presumably due to SP release, which has been shown to trigger a neurogenic, primarily neutrophilic nasal inflammatory response [54,55]. The agents that cause corrosive rhinitis cause nasal inflammation which can break down and ulcerate the nasal mucosa. Lastly, allergic occupational rhinitis is IgE mediated and eosinophils, eosinophilic cationic protein, basophils and tryptase have been found in the nasal lavage fluid of these patients [56-58].

Hormonally induced rhinitis can be seen in pregnancy. It is unclear exactly how changes during pregnancy result in rhinitis. Symptoms of rhinitis may have been present prior to pregnancy but not fully appreciated [59]. An increased circulating blood volume and progesterone induced smooth muscle relaxation may lead to increased nasal mucosal blood pooling and subsequent symptoms of congestion [60]. In addition, high levels of estrogen, prolactin, VIP and/or placental growth hormone have been associated with increased nasal mucosal swelling from increased vascular leakage, glandular secretion and nasal vascular smooth muscle relaxation [61-63].

Rhinitis medicamentosa is believed to be caused not by vasodilation, but interstitial edema [64]. The medications responsible have been shown to cause damage to the nasal mucosa. Histologically, severe epithelial and sub epithelial damage can occur, such as loss of ciliated cells as well as gaps and ruptures of basal lamina. Furthermore, partial hyperplasia of goblet cells and proliferated seromucous glands can be seen [65].

Atrophic rhinitis is divided into primary (idiopathic) or secondary rhinitis. Primary atrophic rhinitis is characterized by nasal mucosal atrophy, dryness, crusting and a foul smelling odor [66,67]. The cause is unknown, but maybe associated directly or indirectly with Klebsiella ozaenae, Staphlococcus aureus, Proteus mirabilis and Escherichia coli from initial infection or secondary infection of already damaged nasal mucosa [68]. Secondary atrophic rhinitis is more commonly due to chronic sinusitis, granulomatous disease, excessive nasal turbinate surgery, trauma or irridation [68]. A summary of the proposed pathogeneses of non-allergic rhinitis is provided in Table 2.

**Table 2:** Types of non-allergic rhinitis and proposed mechanisms

RHINITIS	PROPOSED MECHANISM
**Non-Allergic Rhinitis:**	
Vasomotor Rhinitis	CNS stimulation leading to inhibition of the sympathetic nervous system response and enhancement of the parasympathetic response[33].

Gustatory Rhinitis	Muscarinc receptor stimulation[45].

Infectious Rhinitis	Typically viral or bacterial induced inflammation[20].

Non-allergic rhinitis with eosinophilia syndrome (NARES)	Eosinophilia leading to direct nasal mucosal damage and decreased mucocilliary clearance[48-53].

**Occupational Rhinitis: **[54-58]	
Annoyance	Subjective without evidence of inflammation.

Irritant	Substance P induced neutrophilic inflammation.

Corrosive	Agent directly damages nasal mucosa.

Allergic	IgE mediated.

**Other Rhinitis Syndromes: **[60-63]	
Hormonally induced Rhinitis	Increased circulating blood volume and possible hormonal influences (e.g. estrogen, progesterone) leading to vascular pooling and smooth muscle relaxation causing nasal congestion.

Rhinitis Medicamentosa	Direct mucosal damage by alpha-adrenergic agent causing loss of ciliated cells and interstitial edema[64,65].

**Atrophic Rhinitis: **[66-68]	
Primary Atrophic Rhinitis	Infectious i.e. Klebsiella ozaenae
Secondary Atrophic Rhinitis	Identifiable causes: Chronic sinusitis, turbinate surgery and irradiation

Listed above are some of the proposed mechanisms for the different types of non-allergic rhinitis.

## Differential Diagnosis

As evidenced in the case presented and discussion thereafter, thorough evaluation of rhinitis is critical for proper care. There are many conditions that can mimic both allergic and non-allergic rhinitis. These conditions need to be considered in the differential diagnosis and are listed in Table 3.

**Table 3:** Conditions that Mimic Rhinitis Symptoms

Nasal polyps
Anatomic abnormalities: E.g. Trauma or nasal tumors (Benign or Malignant)

Autoimmune: e.g. Sjogren syndrome, SLE, Relapsing polychondritis, Churg-Straus syndrome or Wegener granulomatosis

Metabolic: e.g. Hypothyroidism or acromegaly.

CSF Rhinorrhea

Primary Ciliary Dyskinesia

Cystic fibrosis

Immunodeficiency

Adapted from Wallace et al [21].

## Concluding Remarks

Rhinitis is a prevalent condition, resulting in direct and indirect costs in the billions of dollars. There are many causes and often the treatments overlap. However, diagnosis can often be unclear and sometimes incorrect. A thorough history and physical exam are vital, and inhalant allergy skin testing is essential to evaluate for allergic etiology.

In the case we present here, the patient's rhinorrhea was twice misdiagnosed as due to allergic rhinitis or VMR, before she sought allergy evaluation. Her CSF rhinorrhea, undiagnosed, could have resulted in serious sequelae such as meningitis and possibly death. As well, it could have been a sign of another underlying pathology such as malignancy or tumor. In her case, we believe the CSF leak was spontaneous, though it is difficult to prove whether prolonged moth ball exposure and resultant toxicity may have played some role.

Non allergic rhinitis encompasses a vast and distinct set of conditions. These conditions differ dramatically in their pathogenesis and can differ in their treatments. Evaluation of rhinitis by an allergy specialist is often necessary to establish the correct diagnosis and treatment regimen.

## Abbreviations

(NAR): Non-allergic rhinitis; (AR): Allergic Rhinitis; (VMR): Vasomotor rhinitis; (CSF): Cerebrospinal fluid; (ICP): intracranial pressure; (NARES): Non-allergic rhinitis with eosinophilia syndrome; (BENARS): Blood eosinophilia-nonallergic rhinitis syndrome; (SP): Substance P; (CGRP): Calcitonin gene-related peptides; (VIP): Vasoactive intestinal peptide; (NPY): Neuropeptide tyrosine; (NO): Nitric oxide; (NOS): Nitric oxide synthase; (NADPH): Nicotinamide adenine dinucleotide phosphate; (NKA): Neurokinin A; (TXA2): Thromboxane A2; (RAST): Radioallergosorbent test.

## Competing interests

The authors declare that they have no competing interests.

## Authors' contributions

CN reviewed the literature and assembled the body of the text, figures, and tables. LMM reviewed the accuracy of the neurosurgical portion of this review. DBL and CM selected the topic of this manuscript and provided critical input into construction of the text, figures and tables. All of the authors shared in extensively editing the manuscript. All authors read and approved the final manuscript.
